# Salmon and Human Thrombin Differentially Regulate Radicular Pain, Glial-Induced Inflammation and Spinal Neuronal Excitability through Protease-Activated Receptor-1

**DOI:** 10.1371/journal.pone.0080006

**Published:** 2013-11-20

**Authors:** Jenell R. Smith, Peter P. Syre, Shaina A. Oake, Kristen J. Nicholson, Christine L. Weisshaar, Katrina Cruz, Robert Bucki, Bethany C. Baumann, Paul A. Janmey, Beth A. Winkelstein

**Affiliations:** 1 Department of Bioengineering, University of Pennsylvania, Philadelphia, Pennsylvania, United States of America; 2 Department of Physiology, University of Pennsylvania, Philadelphia, Pennsylvania, United States of America; 3 Department of Neurosurgery, University of Pennsylvania, Philadelphia, Pennsylvania, United States of America; University of Arizona, United States of America

## Abstract

Chronic neck pain is a major problem with common causes including disc herniation and spondylosis that compress the spinal nerve roots. Cervical nerve root compression in the rat produces sustained behavioral hypersensitivity, due in part to the early upregulation of pro-inflammatory cytokines, the sustained hyperexcitability of neurons in the spinal cord and degeneration in the injured nerve root. Through its activation of the protease-activated receptor-1 (PAR1), mammalian thrombin can enhance pain and inflammation; yet at lower concentrations it is also capable of transiently attenuating pain which suggests that PAR1 activation rate may affect pain maintenance. Interestingly, salmon-derived fibrin, which contains salmon thrombin, attenuates nerve root-induced pain and inflammation, but the mechanisms of action leading to its analgesia are unknown. This study evaluates the effects of salmon thrombin on nerve root-mediated pain, axonal degeneration in the root, spinal neuronal hyperexcitability and inflammation compared to its human counterpart in the context of their enzymatic capabilities towards coagulation substrates and PAR1. Salmon thrombin significantly reduces behavioral sensitivity, preserves neuronal myelination, reduces macrophage infiltration in the injured nerve root and significantly decreases spinal neuronal hyperexcitability after painful root compression in the rat; whereas human thrombin has no effect. Unlike salmon thrombin, human thrombin upregulates the transcription of IL-1β and TNF-α and the secretion of IL-6 by cortical cultures. Salmon and human thrombins cleave human fibrinogen-derived peptides and form clots with fibrinogen with similar enzymatic activities, but salmon thrombin retains a higher enzymatic activity towards coagulation substrates in the presence of antithrombin III and hirudin compared to human thrombin. Conversely, salmon thrombin activates a PAR1-derived peptide more weakly than human thrombin. These results are the first to demonstrate that salmon thrombin has unique analgesic, neuroprotective and anti-inflammatory capabilities compared to human thrombin and that PAR1 may contribute to these actions.

## Introduction

Neck pain is estimated to affect up to 70% of the general adult population, with substantial annual physical burdens, health care costs, and disability [1], [2], [3]. Neuropathic pain is classified as pain resulting from direct injury or inflammation to the nervous tissue, which can become chronic and persist long after an initial injury [4], [5]. The cervical dorsal nerve roots, which are comprised of sensory axons, are especially susceptible to injurious mechanical loading. When the roots are compressed, even transiently, they can produce chronic radiculopathy, which is a pain or numbness that radiates down the arm or leg [6], [7], [8], [9], [10], [11]. Compressive nerve root injuries can occur from geometrical changes in the intervertebral foramen caused by cervical disc herniation, spondylosis and/or spinal trauma [6]. Despite the prevalence and debilitating nature of neck pain, the current understanding of chronic pain and potential mechanisms for providing effective treatment are lacking.

Animal models of nerve root compression have defined many of the neuronal and glial mechanisms that contribute to nerve root-mediated pain. A transient (15-minute) compression applied to the nerve root induces immediate and long-term cellular modifications at the site of injury as well as in the spinal dorsal horn where the injured afferents synapse [8], [12], [13], [14], [15]. Within 1 hour after root compression, the transcription and production of inflammatory cytokines, including IL-1β, TNF-α and IL-6, are increased in the ipsilateral spinal cord [14], [16]. In addition, blocking IL-1 and TNF-α from binding to their respective receptors early after a painful root compression attenuates pain, an effect that is sustained after injury [14], [16]. This early (within hours) inflammatory response subsides within one day but spinal microglia and astrocytes become activated by this time [14], [15], [17]. Glial activation in the spinal cord remains elevated at 1 week after injury; at this same time neurons within the injured nerve root exhibit degeneration and disruption of the axonal transport of neuropeptides such as substance P and CGRP to the dorsal horn [13], [18], [19], [20]. Along this same time course, the neurons within the deep laminae of the spinal dorsal horn become hyperexcitable to mechanical stimulation of the forepaw [21]. Axonal degeneration after painful nerve root compression is accompanied by the infiltration of macrophages at the root, both of which persist for at least 2 weeks when pain is still present [12]. Many of the current pain treatments are ineffective at reducing pain [22], [23], [24], [25]. However, based on the complicated timing of inflammatory and neuronal dysfunction after painful nerve root injury, a treatment that mitigates the early inflammatory response and promotes nerve root health has the potential to attenuate chronic pain after nerve root compression.

Thrombin, a serine protease involved in the coagulation cascade, has been shown also to mediate glial and neuronal responses that contribute to nociception [26], [27], [28], [29]. Yet, thrombin’s role in pain is controversial since this enzyme is capable of both *initiating* and *attenuating* pain depending on its concentration and the site of administration [27], [30], [31], [32]. Human thrombin immediately attenuates the behavioral sensitivity that develops by NMDA stimulus when it is administered intrathecally over a range of low concentrations (1.5×10^−17^–1.5×10^−14^mol/mouse) before the stimulus [31]. But, it also induces sustained mechanical allodynia when given alone at a higher concentration (1×10^−12 ^mol/mouse) [27]. However, when a single injection of human thrombin is given in the rat forepaw it transiently decreases mechanical algesia, or sensitivity to mechanically-induced pain, evoked by a noxious stimuli [30]. In contrast to mammalian thrombin, the *salmon* coagulation factor, fibrin, which is comprised of fibrinogen and thrombin derived from salmon, has been shown to attenuate pain when administered at the root immediately after a painful compression [32]. This same treatment by salmon fibrin also reduces the infiltration of phagocytotic macrophages at the root, supporting its anti-inflammatory role [32]. In comparison to its human counterpart, salmon fibrin promotes more locomotor and bladder function recovery when given after traumatic spinal cord injury without intensifying pain, suggesting that it is also neuroprotective [33]. Although analgesic properties of salmon fibrin have been demonstrated, it is not known if salmon *thrombin* alone can also attenuate pain after a neural trauma and how it affects inflammation and neuronal health and function.

Thrombin initiates cellular responses by enzymatically activating a subset of a family of G-protein coupled receptors known as protease-activated receptors (PARs) [34]. Thrombin is capable of activating PARs 1, 3 and 4, but has the highest affinity for PAR1 due to a hirudin-like sequence of amino acids positioned near the extracellular cleavage site that interacts with an exosite of thrombin [35], [36]. Recently, PAR1 activation, via thrombin or man-made activating peptides, has been implicated as a potential regulator of both pain and inflammation [26], [37], [38], [39], [40], [41], [42]. PAR1 expression has been confirmed on cells resident in the central nervous system, including astrocytes and neurons, as well as in a subset of nociceptive neurons within the DRG [38], [39], [40], [42], [43]. Activation of PAR1 by human thrombin or a PAR1 activating peptide induces an immediate increase in intracellular calcium levels and translocation of PKCε from the cytoplasm to the neuron surface, amplifies the release of calcitonin gene-related peptide (CGRP) in response to heat stimuli and enhances electrical currents in response to TRPV1 activation [40]. The activation of astrocytic PAR1 also contributes to their activation and their production of inflammatory cytokines [28], [29], [44], [45], [46], [47]. PAR1^−/−^ mice exhibit significantly less GFAP expression after a cortical stab wound compared to wild type mice [46], further implicating a role of PAR1 in glial activation. Human thrombin induces the transcription and secretion of IL-1β, TNF-α and IL-6 by cultures of astrocytes as early as 6 hours and for up to 24 hours after administration [44], [45], [47]. Since PAR1 activation by thrombin influences astrocytic inflammation, neuronal electrophysiology and pain, we hypothesize that human and salmon thrombin may activate this receptor differently contributing to possible differences in pain and cellular responses.

The aim of the current study was to test the hypothesis that salmon thrombin has unique analgesic properties for nerve root-mediated pain compared to its human analog in the rat. The study also examines cellular and molecular differences between the two species of thrombin by comparing their effects on nerve root health and spinal neuronal electrophysiology after painful nerve root compression, their induction of early inflammatory cytokine production in mixed glial-neuronal cultures and their proteolytic capability. In order to evaluate the ability of each species of thrombin to attenuate behavioral sensitivity, rats undergoing a painful nerve root compression were immediately treated with either species of thrombin applied directly to the nerve root. Following injury and treatment, mechanical allodynia (i.e. pain produced in response to a normally non-noxious mechanical stimulus) was measured for 7 days, at which point the nerve root was assessed for axonal myelination and infiltration of macrophages and spinal electrophysiology was evaluated. Mixed cultures of astrocytes and neurons derived from embryonic rat brain cortices were used to define if the two species of thrombin differentially control inflammation by measuring IL-1β and TNF-α transcription and IL-6 secretion early after thrombin treatment. Proteolytic differences between salmon and human thrombin were measured using fluorogenic synthetic peptides corresponding to the amino acid sequences of PAR1 and the thrombin substrate, fibrinogen, in the absence and presence of thrombin inhibitors.

## Methods

### Ethics Statement

This study was carried out in accordance with the recommendations in the Guide for the Care and Use of Laboratory Animals of the National Institutes of Health. All animal protocols were in approved by the University of Pennsylvania Institutional Animal Care and Use Committee and adhered to guidelines of the Committee for Research and Ethical Issues of the International Association for the Study of Pain [48]. All surgery was performed under anesthesia, and all efforts were made to minimize suffering.

### Formulation of Human & Salmon Thrombin

Thrombin from human plasma (Sigma Aldrich, St. Louis, MO) lyophilized from a sodium citrate buffer was reconstituted in sterile water to a concentration of 100 U/ml according to manufacturer protocols. For some studies human thrombin standard 467489 from the NIH was obtained from Sea Run Holdings, Freeport, ME. Salmon thrombin (Sea Run Holdings, Freeport, ME) was prepared from precipitates derived from anticoagulated salmon blood, as previously described [32], [49], [50]. Salmon thrombin was reconstituted in a buffer consisting of 1 M NaCl, 1 mM EGTA, 20 mM Tris, pH 7.0 and 0.6 mg/ml sucrose at a concentration of >1000 U/ml [32]. Both thrombin solutions were aliquoted and stored at −80°C until used for testing. The activity of human and salmon thrombin was determined prior to each experiment using either the fluorogenic thrombin substrate III (Calbiochem, San Diego) diluted in PBS or the chromogenic substrate Chromozym TH (Roche, Nutley, NJ) in order to ensure equivalent activity between thrombin species in each study.

### Michaelis-Menton Kinetic Analysis

Various kinetic analyses of salmon and human thrombin were carried out in order to determine if both species of thrombin have the same enzymatic capabilities toward clotting substrates prior to examining their effect on nociceptive mechanisms. The enzymatic activities of salmon and human thrombin towards the cleavage site within the Aαchain of human fibrinogen were measured using the chromogenic peptide substrate Chromozym TH (Roche Applied Science, Indianapolis, IN). An aliquot of human thrombin from a 100 U/ml stock solution was assayed using Chromozym TH following the manufacturer’s instructions. An aliquot of salmon thrombin was then diluted until it produced the same rate of Chromozym TH cleavage as 100 U/ml human thrombin. Reaction rates were determined by measuring the rate of optical density change at 405 nm after dilution of salmon or human thrombin to approximately 0.03 U/ml in 3 mL spectrophotometer cuvettes containing Chromozym TH concentrations ranging from 1.8 µM to 180 µM. Km and Vmax were calculated from Lineweaver-Burk plots using standard methods. Cleavage experiments were carried out at room temperature. The human thrombin stock was measured in triplicate (n = 3) and two different salmon thrombin lots were each measured in triplicate (n = 6). Chromozym TH concentrations were varied at random to avoid any systematic trends that might occur if there were any aging-dependent changes in thrombin activity. All assays were completed within 2 hours of thawing while thrombin was kept on ice. Data were reported as group average for each Chromozym TH concentration ± standard error and differences between groups were determined using two-way analysis of variance (ANOVA) for group and concentration as the variables.

### Human Fibrinogen Clotting Tests of Salmon & Human Thrombin

The ability of salmon and human thrombin to polymerize human fibrinogen was measured using clotting time assays. For each assay, 2 mg/ml human or salmon fibrinogen in PBS was polymerized by addition of salmon thrombin (3 lots run in triplicate, n = 9) or a human thrombin standard 467489 (n = 3) at different concentrations ranging from 8 U/ml to 0.25 U/ml. All clotting experiments were carried out at 37°C. Salmon thrombin activity was normalized to the human thrombin standard using Chromozym TH prior to experiments. Clotting times were measured using previously reported methods of tapping the glass vial containing the reaction mixture every 2 seconds until a gel had formed [51], [52], as evident by lack of movement of an air bubble near the meniscus. Statistical differences for the clotting times between groups for each species of fibrinogen were determined using two-way ANOVAs with group and concentration as the factors.

### Surgical Protocol & Behavioral Assessment

In vivo experiments were performed on male Holtzman rats (Harlan Sprague-Dawley, Indianapolis, IN), weighing 358±26 g at the start of the study. Rats were housed under conditions compliant with the U.S. Department of Agriculture and Association for Assessment and Accreditation of Laboratory Animal Care including a 12−12 hour light−dark cycle and free access to food and water.

Rats receiving a painful nerve root injury underwent a transient compression of the right C7 dorsal nerve root [8], [14], [32]. Briefly, surgical procedures were performed under inhalation anesthesia with the rat in the prone position (4% isoflurane for induction, 2% for maintenance). An incision was made from the base of the skull to the T2 spinous process. Along the dorsal region of the spine, the posterior bones and the facet joint on the right side of the C6/C7 spinal levels were removed in order to expose the right C7 dorsal nerve root. A hole was made in the dura mater and the cervical nerve root was compressed for 15 minutes with a calibrated 10 gram-force microvascular clip (World Precision Instruments, Sarasota, FL). Following clip removal, any blood was cleared from the compressed nerve root and 20 µl of either salmon (STh, n = 13) or human (HTh, n = 11) thrombin (2 U/ml in neurobasal media) was added to the nerve root. A separate control group received a vehicle treatment (NB media, n = 12) of 20 µl of only neurobasal media. A fourth surgical group was included to serve as a surgical control in behavioral studies (sham, n = 5); rats underwent all surgical procedures except for compression of the nerve root. For all studies, wounds were closed with polyester suture and surgical staples. Rats were allowed to recover in room air under continuous monitoring.

Behavioral sensitivity was assessed in the forepaw by measuring mechanical allodynia on days 1, 3, 5 and 7 after injury [8], [32]. Allodynia was also measured for each rat before any surgical procedures to establish baseline responses. Prior to each testing session rats were placed in elevated cages with mesh bottoms and allowed to acclimate for 15 minutes. Mechanical allodynia was measured by stimulating the plantar surface of the forepaw on the side ipsilateral to the injury, using 1.4, 4 and 10 g von Frey filaments (Stoelting Co., Wood Dale, IL). Testing sessions consisted of three rounds of 10 stimulations with each filament to the paw, separated by a 10 minute rest period. A positive response was considered as a paw withdrawal and was often accompanied by licking or shaking of the paw. The number of paw withdrawals in a session were counted for each rat, averaged within groups for each day and reported as the measurement of mechanical allodynia. Separate repeated measures ANOVA with Tukey’s test was used to determine statistical differences between groups overall and on individual days for each testing filament. After behavioral testing on day 7, rats that underwent surgery were divided into two groups and were used for either immunohistochemistry analysis or electrophysiological procedures.

### Tissue Harvest & Immunohistochemistry

In a subset of the rats that underwent surgical procedures nerve root health was assessed using immunohistochemical techniques (NB media n = 7; HTh n = 4; STh n = 8). Briefly, rats were deeply anesthetized with sodium pentobarbital (65 mg/kg) and transcardially perfused with phosphate buffered saline and then 4% paraformaldehyde [12], [21]. The injured nerve root was harvested, post-fixed, cryoprotected in 30% sucrose, and embedded in OCT medium (Triangle Biomedical Sciences; Durham, NC). Nerve root sections (14 µm thick) were incubated overnight at 4°C with the primary antibodies, mouse-anti-myelin basic protein (SMI94 and SMI99 at 1∶1000; Covance; Princeton, NJ) and rabbit-anti-Iba1 (1∶1000; Wako; Richmond, VA) followed by incubation at room temperature with the secondary antibodies, goat-anti-mouse Alexa 488 (1∶250; Invitrogen; Carlsbad, CA) and goat-anti-rabbit Alexa 568 (1∶250; Invitrogen; Carlsbad, CA) [12]. Root sections were imaged on a Zeiss LSM 510 confocal microscope, using matched exposures and settings for all groups for both antibodies.

Images of nerve roots were analyzed separately to assess the degree of organization of myelin basic protein (MBP) and the amount of Iba1. The organization of myelin basic protein (MBP) was used as a general indicator for nerve root health, with more disorganized labeling indicating a more severely injured root as previously described [12]. Images were qualitatively rated by two individuals who were blinded to groups for the extent of MBP disruption using a 4-point scale: (−) striated MBP patterning; (+) mostly striated MBP with some disruption in labeling; (++) mostly disorganized MBP labeling; (+++) highly disorganized MBP labeling with pockets of debris [9], [32]. Iba1 labeling in the nerve root was quantified using a customized densitometry program created in MATLAB that counts the number of pixels in each frame that are above a user-defined threshold [12], [32], [43]. The number of positive pixels was normalized to that expression in normal untreated rats and averaged across groups. Statistical differences between groups were determined using one-way ANOVA with a post-hoc Tukey’s test.

### Electrophysiological Procedures

A separate subset of the injured rats underwent electrophysiological testing on postoperative day 7 to determine the effect of painful root compression treated with salmon thrombin (STh, n = 6 rats), human thrombin (HTh, n = 7 rats) or vehicle (NB media, n = 5 rats) treatment had on neuronal excitability in the C6–C8 deep lamina of the dorsal horn [9], [21], [53]. Anesthesia was induced with 0.45 mg/kg of sodium pentobarbital injected intraperitoneally (i.p.). The anesthesia plane was monitored and maintained using additional pentobarbital injections (5–10 mg/kg i.p.) following any withdraw response to hind paw pinch. Following appropriate level of anesthesia the surgical site was re-opened and the spinal cord was exposed via a C6–C8 laminectomy. The rat was placed supine and a midline neck incision was made to expose the mid-cervical trachea. The trachea was then cannulated to allow for mechanical ventilation with room air at 40–50 breaths/min with a tidal volume of 2.5–3.0 mL (Harvard Small Animal Ventilator Model 683; Harvard Apparatus; Holliston, MA), and the end tidal concentration of CO_2_ was monitored continuously (Capnogard; Novametrix Medical Systems; Wallingford, CT). To minimize respiratory related spinal cord movement during electrophysiological recordings, a right sided thoracotomy was performed using the lateral intercostal approach. Following these procedures the rat was placed in a stereotactic frame using ear bars and a T2 spinous process clamp. Core temperature was maintained between 36 and 37°C using a heating plate with temperature controller and isolated rectal probe (model TCAT-2DF; Physitemp Instruments, Inc., Clifton, NJ). A durectomy was performed overlying the C6–C8 portion of the spinal cord, exposing the bilateral C6–C8 dorsal roots. Using a #11 blade a linear incision in the pia along the posterior intermediate sulcus was made to enable access of the electrode to the substance of the spinal cord. The spinal cord was then bathed in 37°C mineral oil for the duration of the experiment.

Extracellular voltage potentials were recorded continuously using a glass-insulated Tungsten electrode (FHC; Bowdoin, ME). Collected electrophysiological signals were processed with a 60 Hz noise eliminator (Hum Bug; Quest Scientific; North Vancouver, BC) and digitally sampled and stored at a rate of 25 kHz (Micro1401; CED, Cambridge, UK). Neurons located in the deep lamina of the dorsal horn were recorded by lowering the electrode 450–1000 µm on the ipsilateral side below the pial surface using a micropositioner. Mechanosensitive specific neurons were first identified by brushing the ipsilateral forepaw. Once identified, the depth, location of the stimulus on the forepaw and reactive area were recorded. The stimulation protocol was then performed which included applying stimuli to the area of maximal response to the stimuli, with at least 60 seconds of rest between each stimulus application,. The stimulation protocol consisted of the application of; 10 consecutive light brush strokes at 1-second intervals, 5 consecutive 1-second stimulations at 1-second intervals with von Frey filaments (1.4 g, 4 g, 10 g, 26 g) and a noxious pinch by a 60 g clip (World Precision Instruments, Inc.; Sarasota, FL) applied for 10 seconds.

The recordings were spike-sorted using Spike 2 software (CED; Cambridge, UK) to make certain that recordings from only a single neuron were analyzed. The total numbers of spikes were counted during each 1-second stimulus period and the 1-second period immediately following each of the 5 stimulations. The number of spikes occurring in a 2-second interval before each stimulus testing period were counted and subtracted from the number of spikes produced in each 2-second stimulation period. Spike counts were log transformed to account for a positive skew in the spike totals distribution and a normal distribution was verified. Neuronal firing differences between the 3 groups were evaluated over all filaments using a three-way ANOVA accounting for group, filament strength and stimulus number. Post-hoc Tukey’s tests evaluated differences between the groups overall and at each filament strength tested.

### Culture of Dissociated Brain Cortices

Cultures were isolated from embryonic day 18 rat pup brains [54]. The meninges were removed and the remaining cortices were dissected and dissociated at 37°C in neurobasal media (Invitrogen Corp., Carlsbad, CA) with trypsin (0.3 mg/ml; Sigma-Aldrich, St. Louis, MO)+DNase I (0.2 mg/ml; Amersham Biosciences, Piscataway, NJ). After 20 minutes, soybean trypsin inhibitor (0.5 mg/ml; Gibco, Grand Island, NY) was added and the tissue was broken apart by manual pipetting. Cell solutions were centrifuged at 1000 rpm for 5 minutes and the remaining pellet was re-suspended in DMEM with Glutamax (Gibco, Grand Island, NY) supplemented with fetal bovine serum (FBS; Gibco, Grand Island, NY). Cells were filtered through 60 µm and 28 µm Nitex meshes sequentially and plated at a density of 4×10^6^ cells/ml on T75 tissue culture flasks treated with poly-D-lysine (PDL; Sigma Aldrich, St. Louis, MO). Cultures were maintained at 37°C and 5% CO_2_ and the media was replaced every 3–4 days. Mixed cultures were re-plated at a concentration of 2×10^6^ cells/ml onto 60 mm PDL-coated culture dishes at 14 days in vitro (DIV).

### Real Time PCR for Cytokine RNA

Separate mixed cultures were treated with salmon (STh, n = 5) or human (HTh, n = 5) thrombin (1 U/ml) at 20 DIV (day 0). At 4 hours after treatment, cells were washed with PBS and RNA was harvested using Qiagen’s RNeasy mini kit (Qiagen, Valencia, CA). The concentration and quality of the RNA samples were measured using a NanoDrop spectrometer (NanoDrop Technologies, Wilmington, DE). RNA samples (0.5 µg total RNA) were treated with DNTP (Invitrogen, Carlsbad, CA) and random primers (Invitrogen) for 5 minutes at 65°C before they were reverse transcribed according to manufacturer protocols using Superscript III reverse transcriptase (Invitrogen) and the RNase inhibitor, RNaseOUT (Invitrogen), at 50°C for 45 minutes. Synthesized cDNA was used for real-time PCR with specific primer sequences for the pro-inflammatory cytokines, IL-1β (Fwd: 5′-CAC CTC TCA AGC AGA GCA CAG-3′, Rev: 5′-GGG TTC CAT GGT GAA GTC AAC-3′) and TNFα (Fwd: 5′-ATC ATC TTC TCA AAA CTC GAG TGA CAA-3′, Rev: 5′-CTG CTC CTC TGC TTG GT-3′) [14]. Each reaction contained equal amounts of synthesized cDNA, appropriate primers, and SYBR green master mix (Applied Biosystems, Foster City, CA). Real time PCR was performed using an ABI-7300 system (Applied Biosystems) under the following conditions: 50°C for 2 minutes, 95°C for 10 minutes, followed by 40 cycles of 95°C for 15 minutes and 60°C for 1 minute. All samples were run in duplicate and a no-cDNA standard was included for each run. Expression of the target genes were normalized to levels of cyclophilin-A (Fwd: 5′-TAT CTG CAC TGC CAA GAC TGA GTG-3′, Rev: 5′-CTT CTT GCT GGT CTT GCC ATT CC-3′). Gene expression levels in the thrombin treated groups were normalized to levels in untreated controls (UT, n = 8) from each cortical dissociation and reported as the fold-difference compared to normal. Values were averaged within groups and differences between were tested using a one-way ANOVA, with post-hoc Tukey HSD test.

### ELISA for IL-6

Separate primary cultures were treated with salmon (STh, n = 6) or human (HTh, n = 6) thrombin at a range of concentrations and supernatant collected 8 hours later to measure the concentration of IL-6. To evaluate if the release of this cytokine is thrombin concentration dependent, treatments were given at 0.2, 0.5, and 1 U/ml. A control group that did not receive any thrombin treatment was also included (untreated, UT, n = 4). IL-6 concentration was determined using a rat IL-6 ELISA kit (ThermoScientific, Rockford, IL) following manufacturer’s instructions. Concentrations of IL-6 in cell culture supernatants are reported in pg/ml and averaged for each group. Differences between the human and salmon thrombin treated cultures were determined using a two-way ANOVA with post-hoc Tukey’s test.

### PAR1-Based Synthetic Peptide Cleavage

A fluorogenic synthetic peptide substrate was created corresponding to the amino acid sequence at the human PAR1 cleavage site at greater than 85% purity (Abgent, San Diego, CA). The peptide was comprised of a hydroxyl group, the three amino acids terminal to the native receptor’s extracellular cleavage site (Asp-Pro-Arg) and an amido-4-methylcoumarin (AMC) functional group that fluoresces when hydrolyzed from the peptide. The peptide substrate (40 µM) was added to PBS at 37°C and vortexed. Human (HTh, n = 2) or salmon (STh, n = 2) thrombin at 1 U/ml was added to the solution, vortexed and immediately placed in the fluorimeter. The substrate solution was excited at 346 nm and the fluorescent intensity was measured at 446 nm and recorded over a period of 5 minutes. Substrate hydrolysis was reported as the slope of 4 minutes of the intensity versus time data. Differences were determined using a student’s t-test.

A modified PAR1 FRET peptide (PAR1-hir) was also designed to include the hirudin-like amino acid sequence located on a PAR1 exodomain that interacts with one of thrombin’s exosites to facilitate native PAR1 cleavage via thrombin in vivo [35], [55]. The amino FRET peptide was designed as follows: Abz-LDPRSFLLRNPNDKYEPFW(DNP)-CONH_2_ with hydrolysis occurring between L (Leu) and D (Asp)_._ The PAR1-hir FRET substrate (40 µM) was diluted in PBS at 37°C and 1 U/ml of each species of thrombin (n = 4/group) were added, separately, to the solutions. Immediately following thrombin addition, the mixture was excited at 298 nm and the fluorescence at 355 nm was recorded for 60 seconds. All substrate cleavage data are reported as the slope of the intensity-time plot fitted to 20 seconds of linear data. The difference between STh and HTh was determined using a student’s t-test.

### Fluorogenic Protease Activity Assays

The enzymatic activity of human and salmon thrombin over time when added to serum-containing media was quantified using a fluorogenic substrate-based thrombin-specific protease activity assay. Hydrolysis of the fibrinogen-like substrate by proteases produces a fluorescent signal (440–460 nm). Either salmon (STh, n = 3) or human (HTh, n = 3) thrombin (1 U/ml) was added to DMEM with Glutamax supplemented with 5% FBS which was maintained at 37°C throughout the test duration. Samples were taken immediately after adding thrombin (t = 0), every 5 minutes up to 30 minutes, and again at 60 minutes. Each sample was added to a glass cuvette along with 0.15 mg/ml fluorogenic thrombin substrate III (Calbiochem, San Diego, CA). The sample was vortexed and placed in a fluorimeter. Samples were excited at 346 nm and the fluorescence intensity at 446 nm was collected over a 60 second period at 0.5-second intervals. The slope of the linear portion of that plot was quantified by fitting a line to 20 seconds of fluorescent intensity-time data. The fluorescence intensity-time slope at each time point was normalized to the slope at time t = 0 and averaged for each time point. Differences between groups were determined using repeated measures ANOVA with post-hoc Tukey’s test.

The efficiency of antithrombin III (ATIII) at inhibiting thrombin activity was tested. Stocks of human and salmon thrombin were diluted to equal concentrations in PBS and vortexed prior to the addition of ATIII (Hyphen Biomed, Mason, Ohio). ATIII was tested at concentrations of 0, 1.625, 3.225, 6.45, 12.9, 19.35, 32.25 and 45.15 nM in PBS. The cleavage rate of each thrombin was measured in triplicate for each concentration of ATIII and normalized to the rate of cleavage without any ATIII in solution (0 nM). Averages between human and salmon thrombin were compared overall and at each individual ATIII concentration using a two-way ANOVA with post-hoc Tukey’s test.

In order to determine the efficiency of the thrombin inhibitor, hirudin, at blocking the enzymatic activity of either type of thrombin, hirudin (Sigma Aldrich, St. Louis, MO) was added to a thrombin/fibrinogen substrate solution. Testing was performed at 37°C. The fibrinogen-like substrate was diluted with PBS to a final concentration of 0.15 mg/ml. Hirudin was added to the diluted substrate solution at ratios of [thrombin]/[hirudin] of 0.5, 1, 1.5, 2, 2.5 and 3 and vortexed. Human (n = 3 for each ratio) and salmon (n = 3 for each ratio) thrombin (1 U/ml) were added to separate solutions and samples were vortexed and immediately placed in a fluorimeter. Samples were excited at 346 nm and the fluorescence at 446 nm was measured over 60 seconds. Thrombin activity was reported as the slope of 20 seconds of the fluorescent intensity-time data. Values were averaged for each type of thrombin at each ratio; differences between groups were determined using a two-way ANOVA with post-hoc Tukey’s test.

## Results

### Salmon & Human Thrombin Exhibit Similar Enzymatic Activities Towards Coagulation-Related Substrates

**Figure 1:**
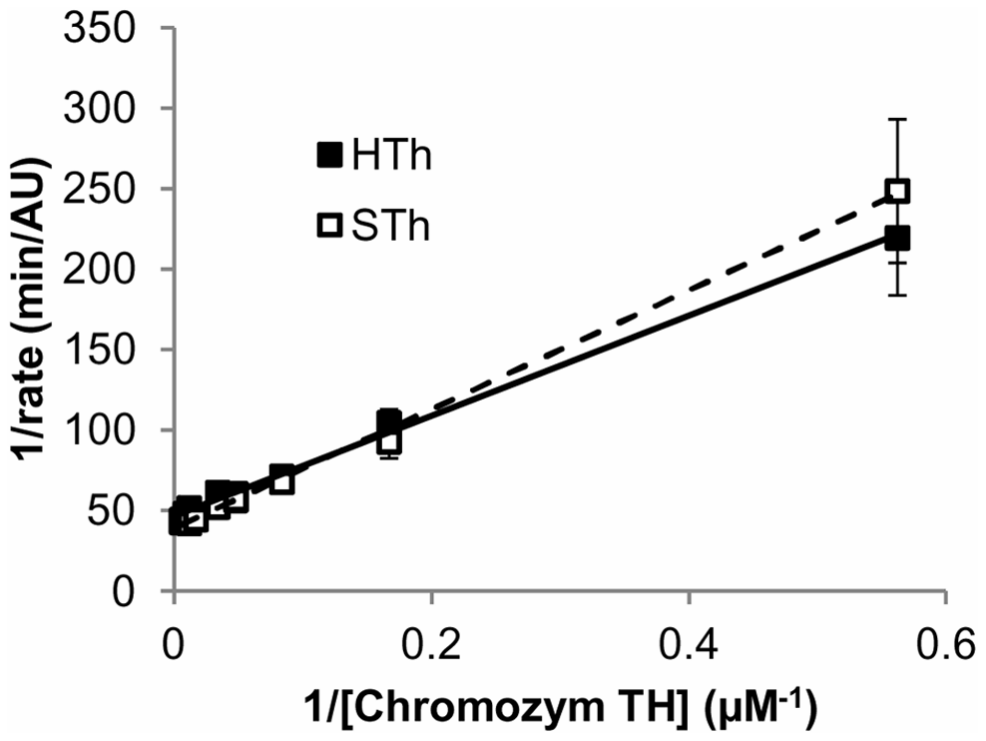
Salmon and human thrombin have the same Michaelis-Menton kinetics towards human fibrinogen Aa chain. The rate of Chromozym TH cleavage by 0.03/ml of thrombin for different concentrations of substrate show no differences in the inverse of cleavage rate between salmon (STh) and human (HTh) thrombin at any concentration. The 1/Km and 1/Vmax of salmon and human thrombin for the chromogenic peptide are derived from the intercept of the X axis at 1/rate = 0 and the intercept of the Y axis at 1/S = 0, respectively. Data are shown as means with standard errors of mean (μ ± SEM).

In order to confirm that salmon and human thrombin have the same enzymatic activity towards coagulation-related substrates prior to any in vivo or in vitro experiments, their capability of cleaving fibrinogen-like peptide substrates and polymerizing both human and salmon fibrinogen were tested. The activity of salmon and human thrombin is indistinguishable when cleaving a chromogenic substrate based on the human Aα fibrinogen chain over a range of substrate concentrations shown by the Lineweaver-Burk plots (Figure 1). The Km values are calculated to be 7.2 µM for human thrombin and 8.5 µM for salmon thrombin, and are within the error of measurement. The Vmax values are indistinguishable for the two species of thrombin.

**Figure 2:**
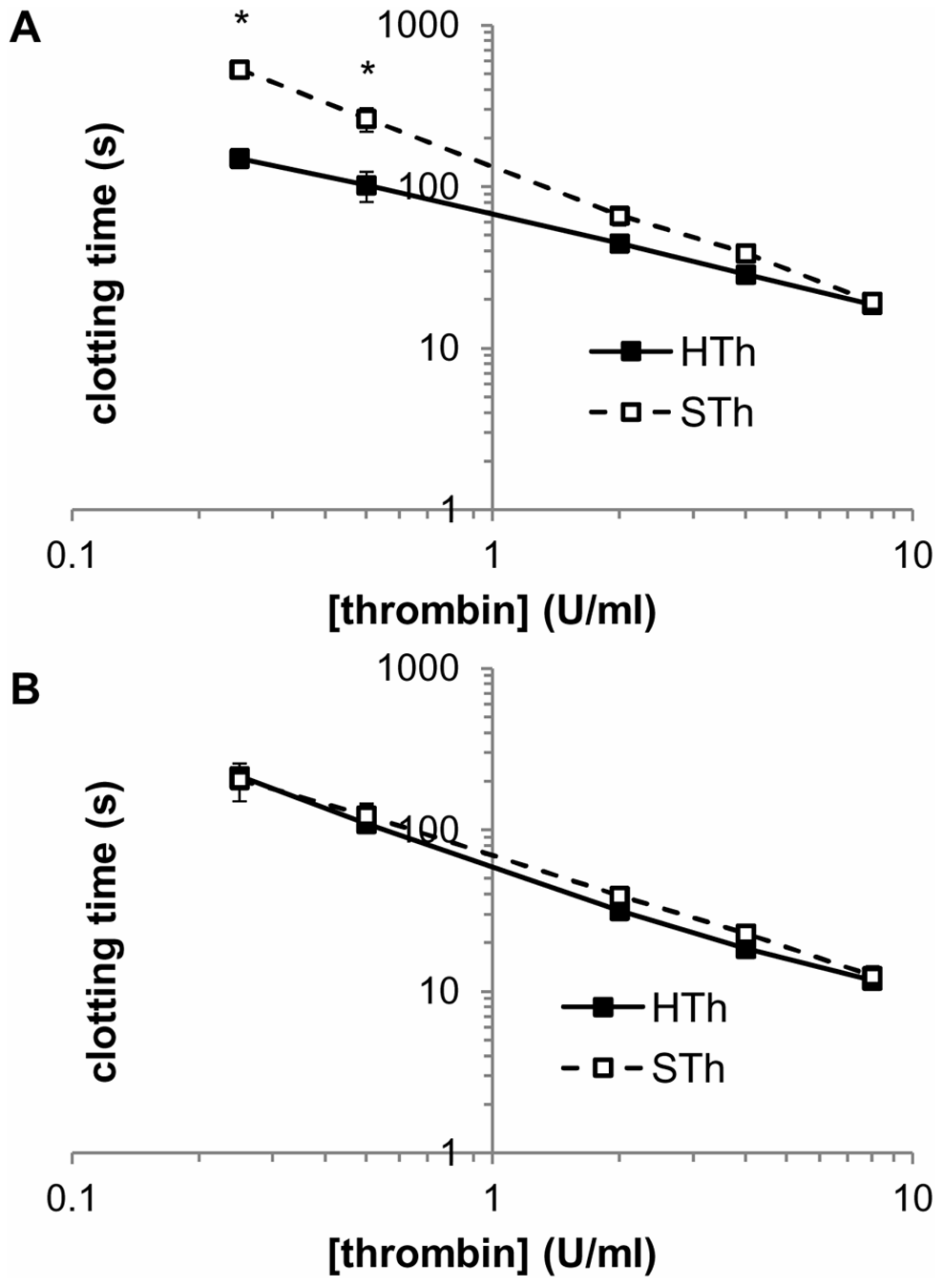
Salmon thrombin clots human fibrinogen slower than human thrombin at lower concentrations. Human thrombin (HTh) or salmon thrombin (STh) (separate assays in triplicate for 3 lots, n = 9) were used to determine clotting times of 2 mg/mg of human (**A**) or salmon (**B**) fibrinogen in PBS. STh clots human fibrinogen at a slower rate than HTh over all concentrations (p<0.001) and at thrombin concentrations of 0.25 and 0.5/ml (*p<0.043). Salmon thrombin and human thrombin have the same clotting rate for human fibrinogen at 2, 4 and 8 U/ml and over the entire concentration range for salmon fibrinogen. Data are shown as means with standard deviations (μ ± SD).

In contrast, salmon and human thrombin differ slightly in their clotting times when administered at lower concentrations. At 2, 4 and 8 U/ml salmon and human thrombin have the same clotting activity when added to human fibrinogen at 37°C (Figure 2A). However, at lower concentrations, clotting times for salmon thrombin are higher than for human thrombin, with significantly differences (p<0.043) at 0.25 and 0.5 U/ml and an overall significant difference (p<0.001) between groups across the range of concentrations (Figure 2A). The difference in polymerization capabilities at the lower concentrations suggests that the activity of the enzymatically active sites of salmon and human thrombin are similar, but that other binding events, such as inactivation of thrombin by human fibrin (i.e. anti-thrombin 1) might be slightly stronger for salmon compared to human thrombin. Conversely, when the clotting tests are performed using salmon fibrinogen, salmon and human thrombin have indistinguishable activities over the range of concentrations tested (Figure 2B). A previous study administered 2 U/ml salmon thrombin in combination with salmon fibrinogen (at 3 mg/ml) and found that treatment to reduce pain after a nerve root compression [32]. Since salmon and human thrombin also exhibit indistinguishable clotting times at that same concentration (Figure 2), 2 U/ml was chosen as the thrombin concentration for the in vivo treatments.

### Behavioral Sensitivity is Attenuated by Salmon Thrombin

**Figure 3:**
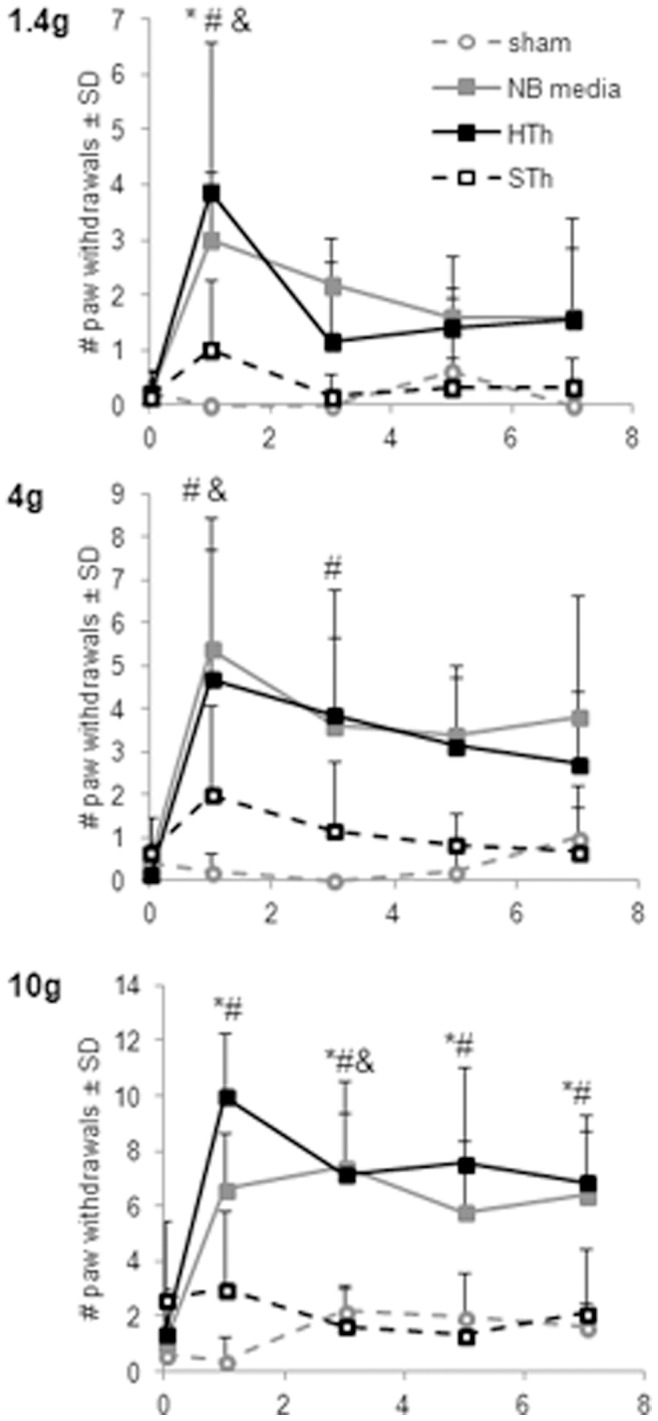
Salmon thrombin attenuates mechanical allodynia after painful nerve root compression in the rat. Mechanical allodynia was significantly elevated in rats after a painful nerve root compression treated with the vehicle, neurobasal media (NB media), compared to sham for all von Frey filament strengths (1.4 g, 4 g, 10 g) over all of the testing days (p<0.012). NB media was also significantly elevated on various individual testing days for various filament strengths (&p<0.036). Human thrombin (HTh) did not alter mechanical allodynia compared to NB media and remained significantly elevated over sham operated rats for all filaments over the entire testing period (p<0.009), on various days for the 1.4 and 4 g filaments (^#^p<0.041) and on each testing day for the 10 g filament (^#^p<0.027). Salmon thrombin (STh) significantly reduced mechanical allodynia compared to NB media (p<0.028) and was unchanged from sham for all filaments over all days. Notably, STh also significantly attenuated allodynia compared to HTh overall (p<0.047), on day 1 for the 1.4 g filament (*p = 0.003) and on each individual testing day for the 10 g filament (*p<0.027). Data are shown as means with standard deviations (μ ± SD).

Behavioral sensitivity was quantified by counting the number of times the rat withdrew the forepaw ipsilateral to the injury in response to stimulation by a series of von Frey filaments over 7 days after injury with treatment of either species of thrombin or the NB media vehicle. Previous studies have reported increased behavioral sensitivity by day 1 after an untreated nerve root compression, which is maintained for at least 7 days [8], [12]. In agreement with those prior reports, nerve root compression treated with NB media induces a significant increase in mechanical allodynia compared to sham for each filament strength over all 7 days (p<0.0126) (Figure 3). Similarly, for rats undergoing nerve root compression and receiving human thrombin, mechanical allodynia is significantly elevated over sham and not different from NB media for all filament strengths (p<0.009) (Figure 3). Interestingly, when a painful nerve root compression is treated immediately with a single administration of salmon thrombin, mechanical allodynia is significantly attenuated compared to both NB media (p<0.028) and human thrombin (p<0.047) for all filament strengths over all days and was not different from sham (Figure 3). Further, for testing with the 10 g filament, salmon thrombin treatment produces significantly fewer paw withdrawals than human thrombin on each testing day (p<0.028) (Figure 3).

### Salmon Thrombin Preserves Nerve Root Health & Attenuate the Local Inflammatory Response

**Figure 4:**
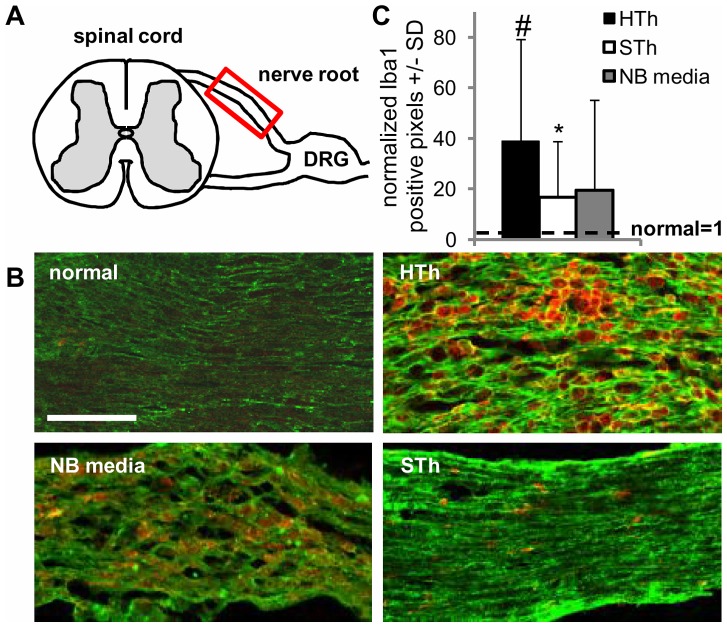
Salmon thrombin preserves nerve root health and prevents inflammation after painful compression in the rat. (**A**) Schematic depicting the spinal cord, nerve root and dorsal root ganglion (DRG); red box indicates location within the nerve root where the root was analyzed. (**B**) Uncompressed nerve roots from un-operated (normal) rats exhibit myelin basic protein (MBP; green) labeling in a striated pattern that is homogenous across the width of the root and no immunoreactivity for macrophages (Iba1; red). On day 7 after a painful root compression treated with neurobasal media (NB media), MBP is disrupted and Iba1 is more abundant. Human thrombin treated roots (HTh) have MBP and Iba1 labeling that is similar to those roots treated with NB media. Salmon thrombin treated roots (STh) exhibit the same striated MBP labeling with minimal Iba1 as normal roots and much less than roots treated with NB media or human thrombin. Scale bar is 100 µm. (**C**) Quantification of positive Iba1 labeling normalized to expression in normal un-operated tissue. Normal tissue exhibits very low levels of Iba1. Human thrombin significantly increases (^#^p<0.001) Iba1 in the nerve root compared to normal. Roots treated with salmon thrombin are not different from normal levels or those treated with neurobasal media, but induces significantly less (*p = 0.035) Iba1 infiltration in the nerve root compared to human thrombin.

Central sensitization can result from injured and/or inflamed afferents, located within an injured nerve root, that synapse in the spinal dorsal horn [4], [56]. Therefore, at day 7 after injury, we examined the disruption of neuronal myelination, as an indicator of root damage. We also assessed the infiltration of macrophages, which contribute to the immune response and engulf excess myelin within the compressed roots (Figure 4A). Under normal conditions, the myelin sheath in the nerve root is intact, as indicated by the smooth striated pattern and there is no-to-minimal labeling for macrophages (Figure 4B; Table 1). At day 7 after painful root compression treated with NB media, the myelin surrounding the primary afferents is less ordered (Figure 4B; Table 1) and there appears to be an increase in macrophages, albeit this increase is not significant (Figures 4B&4C). These findings agree with previous studies demonstrating axonal degeneration, myelin breakdown and macrophage infiltration in the nerve root after a painful nerve root compression at both 7 and 14 days after injury [12], [13], [18]. A painful root compression with human thrombin treatment induced similar disruption in neuronal myelination (Figure 4B; Table 1) and a significant increase (p<0.001) in macrophage infiltration in the root compared to normal tissue (Figures 4B&4C). However, roots treated with salmon thrombin appear much healthier than those treated with NB media or human thrombin (Figure 4B); Iba1 immunoreactivity is significantly less (p = 0.035) than human thrombin treated roots and myelination appears mostly uniform throughout the root (Figures 4B&Table 1). Further, the macrophages in the root after treatment with NB media or human thrombin have a phagocytotic morphology with a globular cell shape surrounding or engulfing myelin debris (Figure 4B). In contrast, those macrophages that infiltrate injured roots treated with salmon thrombin exhibit a resting morphology (Figure 4B), implying that salmon thrombin decreases the severity of the immune response that is initiated after a painful nerve root compression [12].

**Table 1:** Myelin basic protein (MBP) organization in the injured nerve root.

rat ID	MBP rating
**human thrombin**	
T19	+/++
T20	+++
24	+
25	++/+++
**salmon thrombin**	
T1	−/+
T3	+
T4	−/+
T17	++
T18	−
65	+
66	++
67	+
**neurobasal media**	
T5	+++
T6	+
T7	++
T8	++
3	−/+
62	+
63	+
**Normal**	
N1	−
N2	−

−striated MBP patterning;

+mostly striated MBP with some disruption in labeling;

++mostly disorganized MBP labeling;

+++highly disorganized MBP labeling with pockets of debris.

### Evoked Spinal Neuronal Firing Rate is Reduced after Salmon Thrombin Treatment

**Figure 5:**
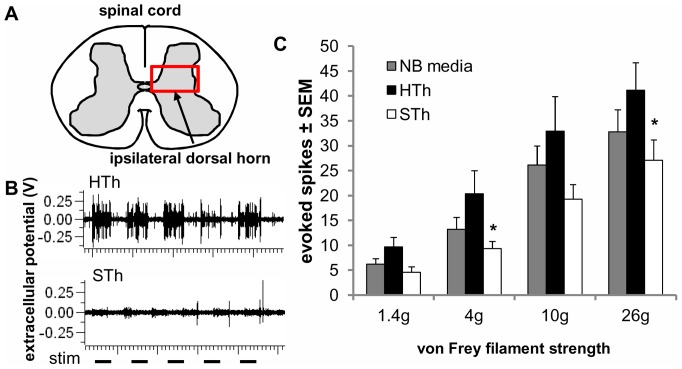
Salmon thrombin reduces spinal neuronal hyperexcitability after a painful root compression in the rat. (**A**) Schematic showing the location of recording in the deep laminae of the ipsilateral spinal dorsal horn. (**B**) Representative extracellular potentials evoked in response to five 1-second stimuli by a 26 g von Frey filament applied to the ipsilateral forepaw showing neurons in the human thrombin (HTh) group are more excitable than those in the salmon thrombin (STh) group. (**C**) Overall, salmon thrombin (STh) produces a significant decrease in the number of evoked spikes compared to both neurobasal media (NB media) (p = 0.002) and human thrombin (HTh) treatment (p<0.001). Salmon thrombin also significantly reduces responses compared to human thrombin in response to stimulation by the 4 and 26 g von Frey filaments (*p<0.029). Interestingly, human thrombin induces a significant increase in evoked firing compared to NB media treated rats overall (p = 0.015). Data are shown as means with standard error (μ ± SEM).

In order to determine if salmon thrombin attenuates neuronal hyperexcitability in the spinal cord in a similar manner to its reduction in allodynia and restoration of nerve root health, neuronal firing was recorded in the ipsilateral spinal dorsal horn at day 7 after injury with each of the treatments. Because evoked neuronal firing rate has been shown to increase in the deep laminae (laminae IV and V) of the dorsal horn at day 7 after this painful nerve root compression in association with sustained behavioral sensitivity [21], we recorded the neuronal response to mechanical stimulation of the ipsilateral forepaw at a depth of 574±102 µm (mean ± standard deviation) in the deep laminae of the dorsal horn (Figure 5A). Neuronal firing rate is significantly reduced when rats are treated with salmon thrombin compared to both NB media (p = 0.002) and human thrombin (p<0.001) over all filament strengths (1.4 g, 4 g, 10 g and 26 g) on day 7 after injury (Figure 5B & 5C). In agreement with the behavioral results (Figure 3), salmon thrombin significantly reduces (p<0.029) the neuronal firing rate evoked by stimulation with the 4 g filament compared to human thrombin treatment (Figure 5). The decrease in firing rate is also evident for stimulation by the 26 g filament (Figure 5). In contrast, there was no difference between groups in evoked firing at individual filament strengths of 1.4 g or 10 g at day 7 (Figure 5), but salmon thrombin did consistently reduce behavioral sensitivity for the filaments over all days tested (Figure 3). Interestingly, human thrombin significantly *increased* (p = 0.015) evoked firing over NB media for stimulation with each of filament strengths (Figure 5), although there was no difference between these groups behaviorally (Figure 3).

### Cytokine Responses are Attenuated in Culture by Salmon Thrombin

**Figure 6:**
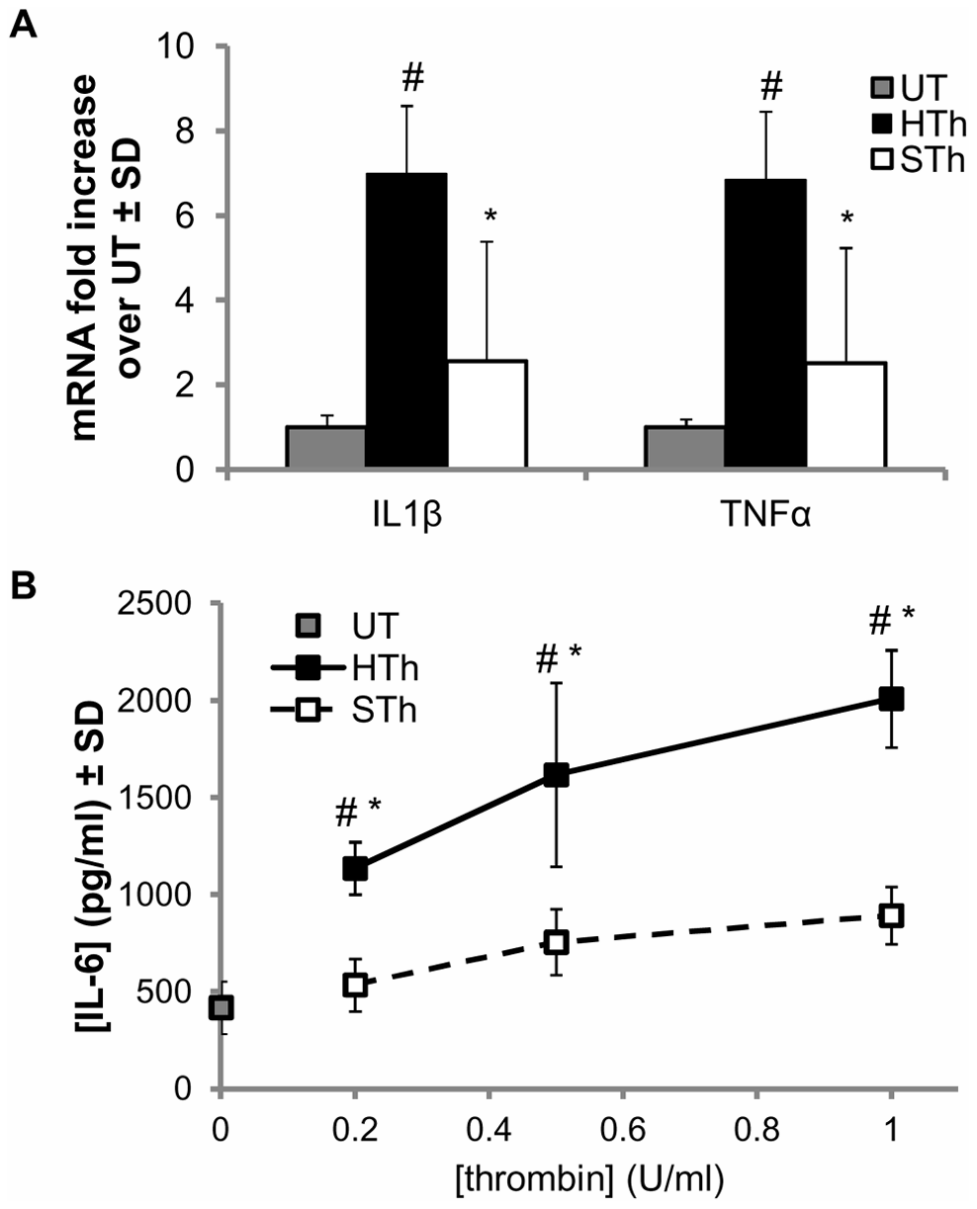
Salmon thrombin does not increase pro-inflammatory cytokines in cortical cultures at early time points. (**A**) IL-1β and TNFα mRNA are significantly upregulated in cortical cultures at 4 hours after their treatment with human thrombin (HTh) at 1 U/ml compared to untreated cultures (UT) (^#^p<0.001). Salmon thrombin (STh), at the same concentration, is unchanged from UT and significantly less than human thrombin treatment (HTh) (*p<0.002). (**B**) IL-6 concentration is significantly greater in cultures treated with human thrombin overall at each individual concentration (^#^p<0.01) for 6 hours compared to the untreated (UT) control. IL-6 secreted from cortical cultures is significantly less after treatment with salmon thrombin (STh) compared to human thrombin (HTh) at 6 hours after stimulation overall (p<0.001) and at each individual concentration of 0.2, 0.5 and 1 U/ml (*p<0.03). Data are shown as means with standard deviations (μ ± SD).

Early upregulation of spinal pro-inflammatory cytokine (IL-1β, TNF-α and IL-6) production has been directly implicated in nerve root-mediated pain, with blocking either IL-1β or TNF-α after painful compression inducing a significant reduction in behavioral sensitivity [14], [16]. For this reason, we measured differences between salmon and human thrombin in their induction and secretion of pro-inflammatory cytokines. In order to distinguish the direct effect of both species of thrombin on inflammatory cytokine production, we used mixed cultures from rat brains containing astrocytes and neurons, the primary cell types of the CNS. IL-1β and TNF-α mRNA is significantly upregulated at 4 hours after treatment with human thrombin compared to untreated controls (p<0.001) (Figure 6A). Another similar increase in pro-inflammatory cytokine transcription by astrocytic cultures has been reported as early as 8 hours after stimulation [45], which supports our findings since the cortical cultures used in our study are primarily comprised of astrocytes. In contrast to human thrombin, those cultures treated with salmon thrombin express IL-1β and TNF-α mRNA at the same levels as untreated cultures and are significantly lower than levels in the cultures treated with human thrombin (p<0.002) (Figure 6A).

To confirm that human thrombin is capable of inducing the secretion of pro-inflammatory cytokines in addition to their transcription, we measured the amount of IL-6 released into the supernatants 6 hours after thrombin stimulation. In agreement with the transcription results at a 1 U/ml treatment dose (Figure 6A), human thrombin induces a significant increase in IL-6 secreted by mixed cortical cultures compared to untreated cultures and salmon thrombin treatment, over a range of low concentrations (p<0.001) (Figure 6B). Cultures treated with salmon thrombin release significantly less IL-6 than untreated cultures (p<0.01) and those treated with human thrombin (p<0.03) at each individual thrombin concentration of 0.2, 0.5 and 1 U/ml (Figure 6B). Further, there is no significant difference between salmon thrombin and untreated cultures at any of the three salmon thrombin concentrations tested. The secretion of IL-6 by cultured astrocytes has been confirmed previously at 20 hours after their stimulation with a higher concentration (20 U/ml) of human thrombin [45].

### Cleavage of PAR1-Like Peptides is Slower by Salmon Thrombin than by Human Thrombin

**Figure 7:**
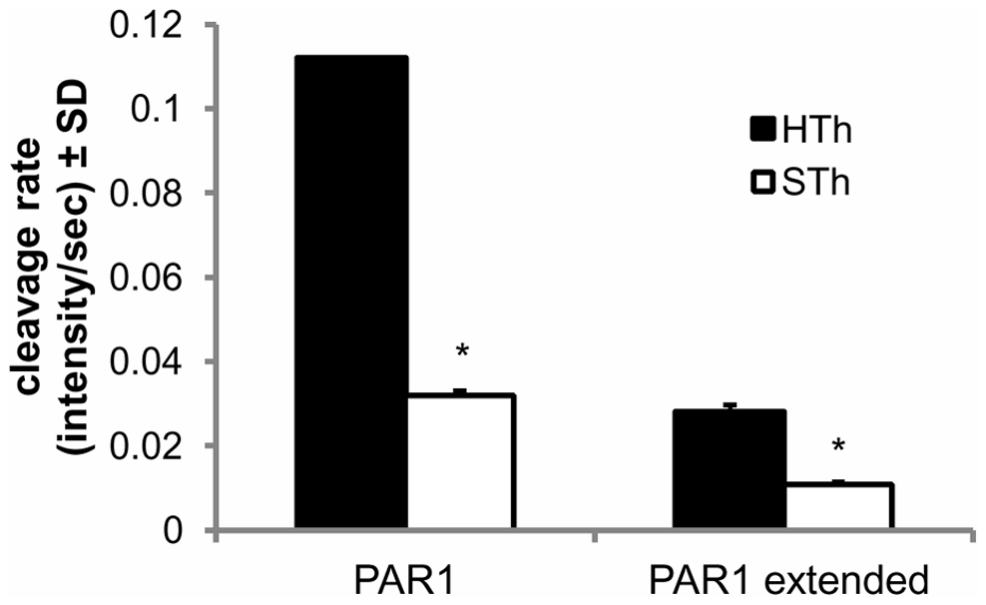
Salmon thrombin cleaves peptides mapping the PAR1 cleavage site slower than human thrombin. Hydrolysis of the fluorogenic PAR1 peptide including only the PAR1 cleavage site (PAR1) is significantly (*p = 0.004) slower for salmon thrombin (STh) than for human thrombin (HTh) (*p = 0.004). For the peptide sequence that also includes a hirudin-like binding site (PAR1 extended), salmon thrombin (STh) also cleaves the peptide significantly slower than human thrombin (HTh) (*p<0.001). Data are shown as means with standard deviations (μ ± SD).

The mechanism by which salmon and human thrombin may induce different cell-controlled outcomes such as allodynia, nerve root health, spinal neuronal hyperexcitability and astrocyte-mediated inflammation is not known. Since PAR1 is the main cellular receptor for thrombin and the degree of its activation, or cleavage, at least partially controls the resulting cellular responses [34], [35], [36], [37], we measured the relative cleavage rate of PAR1-like peptides by salmon and human thrombin in order to examine if PAR1-activation might be responsible for these differences. Salmon thrombin hydrolyzes the fluorogenic PAR1 peptide mapping the cleavage site of PAR1 (Asp-Pro-Arg) at a significantly lower rate than human thrombin does (p = 0.004) (Figure 7). To more accurately mimic the PAR1 receptor, we also created a FRET peptide that included the hirudin-like amino acid sequence on the native receptor that increases the affinity of thrombin to PAR1 [35], [55]. Salmon thrombin also cleaves the PAR1 peptide including the cleavage site and the hirudin sequence (PAR1-extended) at a significantly slower rate than does human thrombin (p<0.001) (Figure 7).

**Figure 8:**
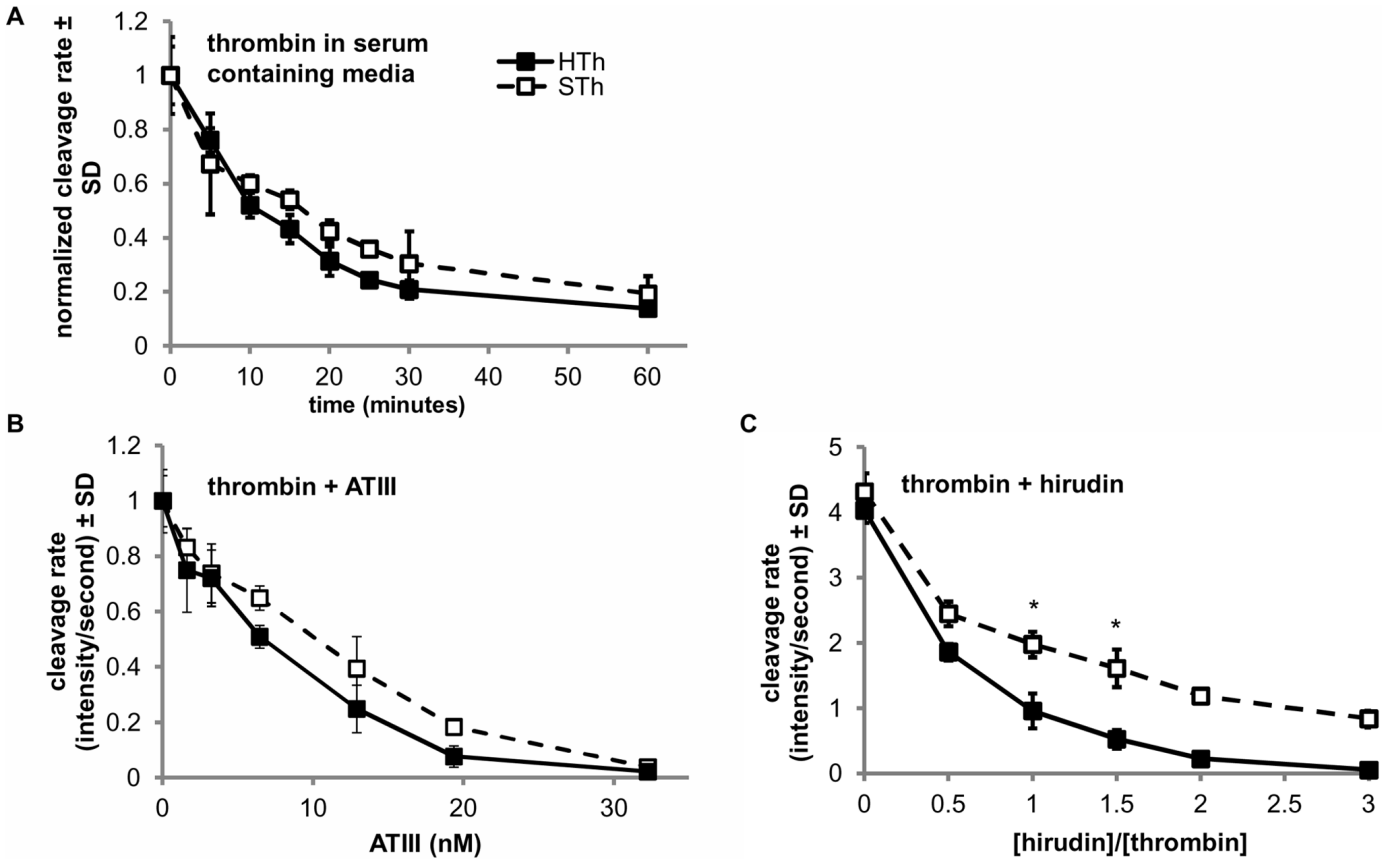
Salmon and human thrombin have similar affinities for serum containing media and ATIII, but not hirudin. (**A**) Salmon thrombin (STh) and human thrombin (HTh) maintain similar enzymatic activity towards a fluorescent fibrinogen-like substrate over time in serum containing media kept at 37°C; their normalized cleavage rate is not different at any time point after thrombin addition. (**B**) The activity of salmon thrombin is inhibited significantly less (p = 0.005) than human thrombin by Antithrombin III (ATIII) overall, but not at any one individual concentration ranging from 0 to 45 nM. (**C**) Salmon thrombin activity towards fibriniogen is inhibited less than human thrombin overall (p<0.001) and at hirudin-to-thrombin ratios of 1 and 1.5 (*p<0.001). Data are shown as means with standard deviations (μ ± SD).

To determine whether the different effects of human and salmon thrombin in vivo might be due to differences in their stability when introduced into the complex injury setting, the enzymatic activities of the two thrombin types were measures as a function of time after introduction to serum-containing cell culture media that are likely to contain some of the proteinases or inhibitors that could inactivate thrombin at the wound site. Both human and salmon thrombin slowly lost activity when added to serum-containing media maintained at 37°C, but there was no difference in the rate at which human and salmon thrombin lost ability to cleave the fluorogenic substrate over a 60-minute time period (Figure 8A). Similarly, when the purified endogenous thrombin inhibitor, antithrombin III (ATIII) is added at concentrations ranging from 1 to 33 nM, there is no difference in effect of ATIII on the cleavage rate of the fibrinogen substrate between the two species of thrombin at any individual concentration (Figure 8B); however, ATIII inhibits the enzymatic activity of salmon thrombin less effectively than human thrombin overall (p = 0.005), indicating that the enzymatic effects of salmon thrombin may last longer than human thrombin at locations where endogenous protease inhibitors are located, such as at injury sites. The exogenous thrombin inhibitor, hirudin, also inhibits salmon thrombin significantly less effectively than human thrombin (p<0.001) as evidenced by salmon thrombin cleaving the fibrinogen substrate significantly faster than human over a range of hirudin to thrombin ratios (Figure 8C).

## Discussion

Despite chronic pain affecting a substantial number of individuals, many of the treatment options are not ideal because they are not effective for all cases, are often accompanied by adverse side effects, including addiction liability and opioid-induced gastrointestinal disorders, among others [22], [23], [24], [25]. Many of these treatments target neuron-driven nociceptive mechanisms within the central nervous system; however, activated glial cells release pro-inflammatory factors and interact with neurons to perpetuate pain states [4], [14], [16], [56], [57], [58], [59], [60], [61], [62], [63]. Therefore, an ideal analgesic targets both the neuronal and inflammatory aspects of pain in concert. Salmon-derived coagulation factors, such as fibrin and thrombin, have been shown to be neuroregenerative, have been hypothesized to be anti-inflammatory under certain circumstances, and salmon fibrin has been reported to have analgesic properties after nerve root trauma [32], [33], [64], [65]. Therefore, we investigated whether or not salmon thrombin exhibits neuroprotective, anti-inflammatory and analgesic properties. The work presented here demonstrates that salmon thrombin induces different behavioral, cellular and kinetic differences compared to its human counterpart.

We show that salmon thrombin given immediately after a painful nerve root compression attenuates pain by day 1 that lasts for at least 7 days after injury; human thrombin does not reduce behavioral sensitivity under the doses and conditions examined. Salmon fibrin has also been shown to reduce pain after nerve root injury for up to 7 days [32], confirming the analgesic capability of multiple coagulation factors derived from salmon. Conversely, mammalian species of thrombin have been correlated to aspects related to the causation of neuropathic pain; inhibition of endogenous thrombin in the central nervous system of the mouse with hirudin reduces sensitivity to mechanical stimuli for up to 6 days after sciatic nerve ligation [27]. The discrepancy in analgesic outcomes for the two species of thrombin has also been observed when human thrombin is administered at different concentrations or anatomic sites [27], [30], [31], [66]. For example, an intraplantar injection of human thrombin increases nociceptive thresholds to heat and mechanical stimuli for only approximately 1 hour [30], [66], whereas a single intrathecal injection of thrombin is sufficient to induce mechanical and heat sensitivity that lasts for up to 9 days [27]. In the current study, both salmon and human thrombin were administered at 2 U/ml, a concentration in which their enzymatic activity toward fibrinogen is not different, suggesting that the different downstream cellular effects produced by these species of thrombin are concentration independent.

At the site of injury, salmon thrombin partially inhibits the infiltration of macrophages and reduces the disruption of axonal myelination, both of which are typically evident at day 7 after injury [12], [13], [18]; this was not observed for human thrombin. Although macrophages often infiltrate regions where demyelination occurs in order to phagocytose myelin debris, their presence and phagocytotic phenotype also indicate a more severe injury [12]. Although we previously showed that salmon *fibrin* inhibits macrophage infiltration at the nerve root at day 7 after a painful compression, axonal demyelination was not evaluated [32]. Here, we show that in addition to attenuating the infiltration of immune cells at the root, salmon thrombin has a neuroprotective effect by preserving axonal myelination after root compression. Although the disorder of myelin within the nerve root is not necessarily a direct measure of neuron integrity, it was used here to help visualize the *extent* of root injury since non-striated myelin patterning corresponds to root degeneration and a decrease in NF200 in this injury model [12], [18], at day 7 when treated with either species of thrombin. Nerve root degeneration, which is accompanied by myelin disruption, develops in this radiculopathy model at some time between day 1 and day 7 but behavioral sensitivity is initiated by day 1 [8], [13], [18], suggesting that nerve root degeneration is not an *initiator* of nerve root pain. Combined with our findings that salmon thrombin attenuates pain by day 1 and protects nerve root health at day 7, salmon thrombin may prevent the *persistence* of root-mediated pain through a neuroprotective mechanism, but other neuronal or glial mechanisms within the root or spinal cord may contribute to its inhibiting development of pain.

This study also shows that spinal neuronal hyperexcitability is reduced by salmon thrombin and is actually increased when treated with human thrombin compared to the vehicle treatment. The electrophysiology data follow the behavioral data; human thrombin and neurobasal media treated injured nerve roots are associated with increased neuronal firing rates and elevated mechanical allodynia compared to salmon thrombin. This suggests that the hyperexcitability of neurons in the spinal cord contribute to pain, which is supported by previous studies showing that different types of painful peripheral tissue injuries alter spinal electrophysiology in the deep dorsal horn [21], [53], [67], [68], [69]. Further, a facet joint injury, which is another spinal injury that is less traumatic to neural tissue than the nerve root injury used here induces spinal neuronal hyperexcitability as early as 1 day when pain is first present [68]. Similar to the painful facet injury, the radicular injury used in this study may also induce changes in spinal electrophysiology earlier than day 7, in which case inhibition of neuronal plasticity in the spinal dorsal horn by salmon thrombin may in fact prohibit the initiation of persistent pain.

In addition to salmon thrombin attenuating neuronal hyperexcitability, we also show that unlike human thrombin, salmon thrombin does not initiate the early production of inflammatory cytokines in neuronal-glial cultures, which may also inhibit pain. Salmon thrombin has no effect on the level of inflammatory cytokines produced by mixed neuronal-glial cultures at early time points (4 and 8 hours) after treatment, whereas human thrombin initiated the production of IL-1β, TNF-α, and IL-6. The pro-inflammatory effects of human thrombin demonstrated in this study are consistent with reports that glial cultures produce IL-1β, TNF-α, IL-6 and a variety of other cytokines by 8 hours lasting for up to 24 hours after stimulation [44], [45], [47], [70]. Increased levels of pro-inflammatory cytokines in vivo leads to the recruitment of glia and their subsequent activation, which can enhance synaptic transmission and amplify nociceptive signaling [62], [71], [72]. In the same radiculopathy model used in this study, mRNAs for pro-inflammatory cytokines are upregulated in the spinal cord and DRG as early as 1 hour after injury and antagonizing TNF-α and IL-1 after injury attenuates allodynia [14], [16]. These early pro-inflammatory responses after nerve root compression lead to increases in glial and immune cell populations in the spinal cord and correlate with sustained behavioral sensitivity [8], [12], [16]. Although the current study only analyzed the transcription on IL-1β and TNF-α, the primary goal was to determine *if* these two species of thrombin differentially activate inflammatory cascades, which is confirmed by the mRNA findings. Future studies are necessary to more completely define the spatiotemporal profile of cytokine mRNA and protein levels, and in response to different thrombin concentrations in order to fully understand the effects of each species of thrombin on neuroinflammation. Yet, these findings do confirm that human thrombin increases the transcription and secretion of pro-inflammatory cytokines that are early regulators of inflammation in vitro, while salmon thrombin does not; it is possible that human thrombin may actually be pro-inflammatory in vivo and be contributing to the production of pain in this model [14], [16].

Our results show that salmon thrombin has a lower affinity than human thrombin for the PAR1 activation site as well as the PAR1 activation site paired with the hirudin-like sequence. This is important when interpreting the molecular mechanisms that distinguish salmon from human thrombin since in many physiological systems, PAR1 activation is the main mechanism through which thrombin alters cell signaling [34], [35]. Through PAR1 activation, thrombin can even initiate *dual* signaling cascades based on the degree of PAR1 activation and possibly even the type of G-protein coupled to the activated receptor [73], [74]. For example, PAR1 activation by thrombin in endothelial cells can induce vascular protection or vascular leakage depending on which sphingosine 1-phosphate (S1P) receptor PAR1 is coupled to, S1P_1_ or S1P_3_ [74]. Within the nervous system, PAR1 agonist concentration also seems to be important for the subsequent signaling; low concentrations of PAR1 activating peptide decrease levels of phosphorylated ERK (pERK), a marker of cellular trauma, whereas high concentrations increase pERK expression, suggesting that high concentrations may amplify effects of trauma [75], [76]. Since salmon thrombin activates the synthetic PAR1 peptides at a slower rate than human thrombin, it is possible that salmon thrombin signals through PAR1 at the nerve root and initiates neuroprotection, whereas higher levels of PAR1 activation by human thrombin may exacerbate injury. Indeed, we show that treated nerve roots treated with salmon thrombin after their compression exhibit less demyelination than the roots with human thrombin, which appear even more disrupted than the control (neurobasal media) treated roots. Although PAR1 cleavage activity is different between salmon and human thrombin, it is possible that other receptors also are partially responsible for differences experienced by these species of thrombin. For example, PAR4 activation has been linked to nociception with an intraplantar injection of PAR4 activating peptide increasing the nociceptive threshold to heat and mechanical stimulation in the rat [77]. Since thrombin is capable of activating PAR4, albeit with a lower affinity than PAR1, it is possible that salmon thrombin activates PAR4 more readily than human thrombin does, also contributing to its analgesic properties and should be explored in future studies.

In contrast to the differences in PAR1 activation rate, our data confirm previous reports that salmon and human thrombin are equally efficient at cleaving the peptide mapping the human fibrinogen cleavage sequence [49], [78] and in clotting fibrinogen at the concentration (2 U/ml) used in this study. Since both species of thrombin cleave the fibrinogen-like peptide with equal affinity, we studied the ability of various thrombin inhibitors at blocking the enzymatic activity of thrombin toward the fibrinogen peptide. Although using a peptide substrate mapping the rat fibrinogen cleavage site may be more applicable to this injury model in the rat, the commercially available three-peptide fluorogenic thrombin substrate III mapping the human sequence was used since rat and human fibrinogen have a very similar peptide structure in the three amino acids before the cleavage site. We show that salmon thrombin is inhibited less effectively by antithrombin III and hirudin over various concentrations. The hirudin inhibition data also support our findings that human thrombin cleaves the PAR1-like peptide with the hirudin-like sequence at a faster rate than salmon thrombin. Protease inhibitors are often present in high concentration at areas of traumatic injury and since human thrombin is inhibited more effectively than salmon thrombin, it is possible that salmon thrombin maintains its enzymatic activity for longer than do human or other mammalian thrombins. Taken together with the PAR1 cleavage data, salmon thrombin may retain its enzymatic activity towards coagulation substrates which would help the wound healing process but would still maintain a low affinity for PAR1 activation which is associated with anti-nociceptive and anti-inflammatory signaling pathways. The findings that both species of thrombin may interact differently with PAR1 and other coagulation substrates are not surprising since the amino acid structures of the two enzymes are different, especially at putative exosites and other regions distinct from the proteolytic site for fibrinogen [79].

Studies inhibiting the activation of PAR1, and even PAR4, after nerve root injury would provide further insight about whether these receptors contribute to the analgesic properties of salmon thrombin. Nonetheless, the results from this study highlight the analgesic, neuroprotective and anti-inflammatory properties of salmon thrombin compared to human thrombin and support its being an ideal early treatment for neuropathic injuries that lead to chronic pain. Our study further implicates PAR1 activation as being a possible contributor to the analgesic and anti-inflammatory actions induced by salmon thrombin. This study provides a foundation for future investigations into the role of PAR1 activation in neuropathic pain and highlight salmon thrombin as a potential novel therapeutic.
